# Miscellaneous London Hospitals

**Published:** 1919-12-20

**Authors:** 


### MISCELLANEOUS LONDON HOSPITALS.

### MORE CHAIRMEN'S REPORTS.

**BROMPTON HOSPITAL.**

(*a*) Immediate Needs.

Increased income to meet current expenditure.

(*b*) Needs for 1920.

Donations towards extending the Frimley Sanatorium to provide for the needs of the ex-sailor and ex-soldier.

(*Signed*) FREDERICK WOOD, *for Chairman.*

**(INCORPORATED) CANNING TOWN WOMEN'S SETTLEMENT HOSPITAL, Plaistow.**

(*a*) Immediate Needs.

£1,558 to clear off the bank overdraft.

(*b*) Needs for 1920.

(1) Increased annual subscriptions of about £700 a year; (2) The building of a new ward for children.

(*Signed*) HUGH KEMSLEY, *Treasurer.*

**CHELSEA HOSPITAL FOR WOMEN.**

(*a*) Immediate Needs.

£3,000 to clear off overdraft at bank.

(*b*) Needs for 1920.

£2,000 beyond ordinary income for maintenance; £14,500 to complete provision for nurses' home whereby forty beds in hospital now used for nursing and domestic staffs will be made available for patients.

(*Signed*) F. W. R. FRYER, *Chairman.*

**CITY OF LONDON HOSPITAL FOR DISEASES OF THE CHEST, Victoria Park, E.**

(*a*) Immediate Needs.

£5,500 to meet apparent deficiency on the maintenance fund.

(*b*) Needs for 1920.

£12,000, amount needed beyond fairly reliable sources of income, for maintenance; £25,000 for completion of urgent structural improvements and extensions delayed owing to the war.

(*Signed*) A. KAYE BUTTERWORTH, *Chairman.*

**FLORENCE NIGHTINGALE HOSPITAL FOR GENTLEWOMEN, 19 Lisson Grove, N.W. 1.**

(*a*) Immediate Needs.

£1,000.

(*b*) Needs for 1920.

Annual subscriptions of £1,000.

(*Signed*) CARDWELL B. BRIDGEMAN, *Chairman.*

**HENDON COTTAGE HOSPITAL ("King Edward Memorial").**

(*a*) Immediate Needs.

Funds to meet additional upkeep entailed by erection of Army hut to provide for waiting list of forty.

(*b*) Needs for 1920.

Enlargement of the permanent building by doubling both the existing wards and making other extensions, including a sitting-room for the staff, an *x*-ray room, and a better operating theatre; estimated cost, £6,000. It is proposed to deal with this in sections, and to start by extending one of the wards during the coming year.

(*Signed*) JAMES BARBER, *Chairman.*

**THE HOSPITAL FOR EPILEPSY AND PARALYSIS, &c., Maida Vale, W.**

(*a*) Immediate Needs.

£2,000 to enable the hospital to finish the year free of debt. It has been so for upwards of forty years.

(*b*) Needs for 1920.

(1) £10,000 for upkeep; (2) £2,000 for re-decoration; (3) £10,000 for extension of out-patients' department; (4) £1,000 for new psychological department; (5) £25,000 for research department.

**HORNSEY COTTAGE HOSPITAL.**

(*a*) Immediate Needs.

Children's ward extension, for which £20,000 is required.

(*Signed*) JNO. A. RITCHIE, *Chairman.*

**LONDON JEWISH HOSPITAL.**

(*a*) Immediate Needs.

£2,000 to complete payment of building in course of construction.

(*b*) Needs for 1920.

£40,000 to finish construction of front block and make a complete hospital with fifty to sixty beds.

(*Signed*) A. GOODMAN LEVY, *Chairman.*

**LONDON LOCK HOSPITAL, W. 9.**

(*a*) Immediate Needs.

£5,814 to clear off overdraft at the bank.

(*b*) Needs for 1920.

£60,000 to build the new wing to the Female Hospital for pregnant women and their infants.

(*Signed*) H. EASON, *Secretary.*

**LONDON SKIN HOSPITAL, 40 Fitzroy Square, W. 1.**

(*a*) Immediate Needs.

Additional baths for treatment purposes.

(*b*) Needs for 1920.

New apparatus and renovation of existing *x*-ray equipment.

(*Signed*) HOWARD SPAIN, *Chairman.*

**MILDMAY MISSION HOSPITAL, BETHNAL GREEN.**

(*a*) Immediate Needs.

New surgical instruments and clock for children's ward.

(*b*) Needs for 1920.

A larger and more up-to-date operating theatre; a laboratory for research work; a new mortuary; more adequate provision for escape in case of fire; electric lighting; increased accommodation for nursing staff.

(*Signed*) (MISS) M. A. BELLAMY, *Deputation Secretary.*

**MILDMAY MEMORIAL HOSPITAL.**

(*a*) Immediate Needs.

£30,000 to reconstruct the hospital so as to adapt it for the treatment of the professional and middle classes of limited means, who will be received at an inclusive fee of 2½ guineas a week, or less in special circumstances. It is hoped to provide 110 beds, and that an average payment of 2½ guineas will make the hospital self-supporting.

(*Signed*) W. S. PENNEFATHER, *Chairman.*

### MISCELLANEOUS LONDON HOSPITALS: MORE CHAIRMEN'S REPORTS.

### MOUNT VERNON HOSPITAL FOR CONSUMPTION AND DISEASES OF THE CHEST, Northwood, Middlesex.

(*a*) Immediate Needs.

£4,000 to repay loans.

(*b*) Needs for 1920.

An additional £6,000 to pay expenses.

(*Signed*) W. J. MORTON, *Secretary.*

### NATIONAL HOSPITAL FOR DISEASES OF THE HEART, Westmoreland Street, W. 1.

(*a*) Immediate Needs.

Funds to provide new installation of instantaneous *x*-ray apparatus.

(*b*) Needs for 1920.

Extension of out-patient department.

(*Signed*) WHELBY E. WHITNEY, Captain, *Secretary.*

### NATIONAL HOSPITAL FOR THE PARALYSED AND EPILEPTIC (Albany Memorial), Queen Square, W.C.

(*a*) Immediate Needs.

Increased revenue of £8,000.

(*Signed*) G. H. HAMILTON, Secretary.

### PADDINGTON GREEN CHILDREN'S HOSPITAL, W. 2.

(*a*) Immediate Needs.

£1,600 to clear off overdraft to bank.

(*b*) Needs for 1920.

£5,000 estimated deficit for 1920.

(*Signed*) NIGEL HANBURY, *Treasurer.*

### PASSMORE EDWARDS HOSPITAL FOR WILLESDEN.

(*a*) Immediate Needs.

A new hospital as Willesden's war memorial, at an estimated cost of £100,000.

(*b*) Needs for 1920.

£85,000 towards the above, the balance being already subscribed or promised.

(*Signed*) H. EDGELL BROWN, *Secretary.*

### QUEEN CHARLOTTE'S HOSPITAL, Marylebone, N.W.

(*a*) Immediate Needs.

£10,000 for maintenance (debt accumulated during the past five years).

(*b*) Needs for 1920.

About £7,000 beyond anticipated receipts for maintenance, and upwards of £50,000 for urgent alterations and improvements.

(*Signed*) ARTHUR WATTS, *Secretary.*

### QUEEN'S HOSPITAL FOR CHILDREN, Bethnal Green.

(*a*) Immediate Needs.

£10,000 to pay off deficit.

(*b*) Needs for 1920.

Increased income to provide for growth in work, and for higher costs due to present conditions.

(*Signed*) T. GLENTON KERR, *Secretary.*

### ROYAL DENTAL HOSPITAL OF LONDON, 32 Leicester Square, W.C. 2.

(*a*) Immediate Needs.

£3,386 to pay off overdraft at bank, tradesmen's accounts outstanding, and instalment due to Prudential.

(*b*) Needs for 1920.

£95,000 to increase hospital accommodation and clear mortgage on present hospital.

(*Signed*) W. J. WADHAM, *Secretary.*

### ROYAL WATERLOO HOSPITAL (FOR WOMEN AND CHILDREN, S.E. 1.

(*a*) Immediate Needs.

£2,000 to £3,000 to meet the deficit on the General Fund.

(*b*) Needs for 1920.

(1) Largely increased donations to meet not only the present-day increase of prices, but to maintain two temporary nurses' homes in Stamford Street, the present home adjoining the hospital having been condemned; (2) General support to the Building Fund to build that portion of the institution which is no longer habitable.

### ROYAL WESTMINSTER OPHTHALMIC HOSPITAL.

(*a*) Immediate Needs.

Funds to meet rising cost of maintenance.

(*b*) Needs for 1920.

Increased accommodation, especially for in-patients.

(*Signed*) J. H. JOHNSON, *Secretary.*

### ST. ANDREW'S HOSPITAL, Dollis Hill, N.W. 2.

(*a*) Immediate Needs.

Reduction of building debt, £4,749; reduction of probable deficit of about £1,300 on current account owing to heavy outlay for repairs, etc., after the soldiers left.

(*b*) Needs for 1920.

Extinction of debt; completion of buildings.

(*Signed*) W. E. CARTON WIART, *Chairman.*

### ST. MARK'S HOSPITAL, City Road, E.C. 1.

(*a*) Immediate Needs.

£4,000 to pay off loan and settle outstanding tradesmen's accounts.

(*b*) Needs for 1920.

£6,000 from voluntary contributions for ordinary expenditure; £1,000 for new nurses' bedrooms.

(*Signed*) A. W. OXFORD, *Chairman.*

### SAMARITAN FREE HOSPITAL FOR WOMEN.

(*a*) Immediate Needs.

£2,000 to pay off bankers' loan.

(*b*) Needs for 1920.

Funds to prevent closing of two of the smaller wards, which otherwise is a matter of certainty.

(*Signed*) GEO. HAWKINS, *Secretary.*

### SOUTH EASTERN HOSPITAL FOR CHILDREN, Sydenham, S.E. 26.

(*a*) Immediate Needs.

£1,000 to carry on with over and above subscriptions.

(*b*) Needs for 1920.

£1,000 for installation of central heating as ordered by King Edward's Committee; £1,200 to erect and equip laundry; £1,500 much needed to enlarge out-patients' department.

(*Signed*) WALTER MASON, *Hon. Secretary.*

### VICTORIA HOSPITAL FOR CHILDREN.

(*a*) Immediate Needs.

£2,000 to meet deficit; £6,000 for central heating; £6,000, extra bedrooms for nurses.

(*b*) Needs for 1920.

Increased income. (*Signed*) H. G. EVERED, *Secretary.*

### WEST END HOSPITAL FOR NERVOUS DISEASES.

(*a*) Immediate Needs.

Deficit maintenance account during 1919, £2,000.

(*b*) Needs for 1920.

Maintenance: Income additional to 1919, £3,000; replace stocks sold to enable us to reconstruct, £9,400 (£8,000 of which *has* to be replaced); reconstruction of out-patients' department and in-patients' department, £5,000.

### WESTERN OPHTHALMIC HOSPITAL.

(*a*) Immediate Needs.

The hospital is now free from debt.

(*b*) Needs for 1920.

£2,500 for the year's maintenance; about £50,000 to rebuild in-patients' department.

(*Signed*) W. BURLEIGH, *Hon. Secretary.*

